# Nickel@Siloxene catalytic nanosheets for high-performance CO_2_ methanation

**DOI:** 10.1038/s41467-019-10464-x

**Published:** 2019-06-13

**Authors:** Xiaoliang Yan, Wei Sun, Liming Fan, Paul N. Duchesne, Wu Wang, Christian Kübel, Di Wang, Sai Govind Hari Kumar, Young Feng Li, Alexandra Tavasoli, Thomas E. Wood, Darius L. H. Hung, Lili Wan, Lu Wang, Rui Song, Jiuli Guo, Ilya Gourevich, Feysal M. Ali, Jingjun Lu, Ruifeng Li, Benjamin D. Hatton, Geoffrey A. Ozin

**Affiliations:** 1 College of Chemistry and Chemical Engineering, Taiyuan University of Technology, Taiyuan, Shanxi 030024 P. R. China; 2 Materials Chemistry and Nanochemistry Research Group, Solar Fuels Cluster, Department of Chemistry, University of Toronto, 80 St. George Street, Toronto, ON M5S 3H6 Canada; 3 State Key Laboratory of Silicon Materials and School of Materials Science and Engineering, Zhejiang University, Hangzhou, Zhejiang 310027 P. R. China; 4 Karlsruhe Institute of Technology (KIT), Institute of Nanotechnology (INT), Hermann-von-Helmholtz-Platz 1, Building 640, Eggenstein-Leopoldshafen, 76344 Germany; 5 Department of Materials Science and Engineering, University of Toronto, 184 College Street, Toronto, ON M5S 3E4 Canada

**Keywords:** Heterogeneous catalysis, Solid-state chemistry, Materials for energy and catalysis, Nanoparticles

## Abstract

Two-dimensional (2D) materials are of considerable interest for catalyzing the heterogeneous conversion of CO_2_ to synthetic fuels. In this regard, 2D siloxene nanosheets, have escaped thorough exploration, despite being composed of earth-abundant elements. Herein we demonstrate the remarkable catalytic activity, selectivity, and stability of a nickel@siloxene nanocomposite; it is found that this promising catalytic performance is highly sensitive to the location of the nickel component, being on either the interior or the exterior of adjacent siloxene nanosheets. Control over the location of nickel is achieved by employing the terminal groups of siloxene and varying the solvent used during its nucleation and growth, which ultimately determines the distinct reaction intermediates and pathways for the catalytic CO_2_ methanation. Significantly, a CO_2_ methanation rate of 100 mmol g_Ni_^−1^ h^−1^ is achieved with over 90% selectivity when nickel resides specifically between the sheets of siloxene.

## Abstract (web summary)

There is a strong push to develop new catalysts and supports to convert low-value CO_2_ into high-value CH_4_. Here, authors found that the internal or external confinement of Ni on multi-layered siloxene supports determined the reaction pathway, activity, selectivity, and stability in CO_2_ methanation.

## Introduction

Carbon dioxide (CO_2_) is a well-known greenhouse gas that can also be used as a zero- or even negative-cost carbon feedstock in the production of valuable chemicals and fuels^1,2^. This technology can be employed in the power-to-gas process, which consists of an initial hydrogen production step via electrolysis of water, followed by the reduction of CO_2_ via the Sabatier reaction (i.e., methanation). This process of converting CO_2_ into methane (CH_4_) is among the most attractive and promising technologies for transforming electrical energy into renewable chemical energy^3–5^. In doing so, it is possible to circumvent the considerable difficulties associated with hydrogen storage, transport, and large-scale utilization. In addition, CH_4_ offers several benefits over hydrogen, as it is easily liquefied, can be more safely stored and transported, and is widely used in energy-intensive applications as a major component of natural gas.

For the catalysis of the CO_2_ methanation reaction, earth-abundant and relatively low-cost Ni metal is an ideal candidate, which has led to its use being intensively explored. As the methanation reaction is highly exothermic, great effort has been devoted to anchoring or immobilizing Ni nanoparticles onto support materials in attempts to alleviate high-temperature sintering^6–9^. Because of such efforts, spatial confinement has emerged as an effective and unique way to limit the growth of Ni nanoparticles^10,11^. Chen et al.^11^ pointed out that a “cage” of mesoporous SBA-16 could confine small Ni nanoparticles, thereby contributing to their high activity in the reverse water–gas shift reaction (i.e., hydrogenation of CO_2_ to CO). However, the design and fabrication of Ni catalysts with high selectivity toward CH_4_ production, rather than the competing reverse water–gas shift reaction, remains a major challenge. In addition, the reaction mechanism for Ni catalysts is still largely ambiguous, both under laboratory conditions and in industrial applications.

Only recently have theoretical and modeling studies begun to show some evidence regarding the CO_2_ methanation mechanism^12–14^. Heine et al.^14^ used ambient-pressure X-ray photoelectron spectroscopy (XPS) to probe the reaction pathways for CO_2_ methanation on a Ni (111) surface. In this system, CO_2_ first dissociated to CO and atomic oxygen at the Ni surface, whereupon CO then decomposed to atomic carbon (*C) before finally being converted to CH_4_ via hydrogenation. This reaction pathway inevitably yields significant quantities of CO and may lead to catalyst poisoning via carbonization (coking) of the catalyst surface.

In this context, a superior Ni catalyst must have high activity and selectivity toward CH_4_ production, as well as good stability under operating conditions. A unique pathway for sustainable CH_4_ production must therefore be achieved by designing the chemistry and structure of not only Ni itself but, more importantly, the supporting materials used to accomplish the desired surface engineering and spatial confinement.

Silicon, being the second most earth-abundant element, is clearly deserving of investigation for such use. Aside from mesoporous silica, which could supply the aforementioned two-dimensional (2D) or three-dimensional (3D) confinement effect^15–17^, and some reports on metal on porous Si^18–21^, the most widely used form of Si for supporting catalysts is SiO_2_ particles, without further chemical or structural design. SiO_2_-supported Ni catalysts have relatively low selectivity toward CH_4_ production (lower than 80%) with the remaining ca. 20% selectivity being toward CO^22^. In this regard, various promoters were added to improve the CH_4_ production selectivity of the Ni/SiO_2_ catalysts^23–25^. In addition, production selectivity could be switched from CO to CH_4_ on Ni/SiO_2_ catalysts by increasing the Ni loading from 0.5 wt% to a much higher value of 10 wt%^26^. However, it is still a challenge to design silicon-supported Ni catalysts with a distinctive structure and superior selectivity for CO_2_ methanation. In an era where 2D catalyst materials are both in great demand and showing significant promise scientifically and technologically, 2D silicon nanosheet materials have historically been overlooked. Only a few reports for its use in catalysis can be found, exemplifying its use in water splitting^27^, ethylene polymerization^28^, formic acid decomposition^29^, 4-nitrophenol reduction^30^, and butene isomerization^31^. In this report, we employ 2D silicon surface chemistry to design catalysts consisting of Ni on siloxene nanosheets (SiXNS), in order to address the challenges posed by CO_2_ methanation. Hydride- and hydroxyl-terminated layered silicon was chosen as the starting material for designing and constructing the uniquely supported Ni nanostructures used in this study. It is shown herein that Si–H hydrolysis can be triggered or avoided during synthesis by using a water- or ethanol-based Ni^2+^ precursor solution, respectively. This strategic choice also determines whether Ni nanoparticles are obtained on the interior or exterior of the SiXNS. The Ni nanoparticles confined inside the SiXNS exhibit improved catalytic activity and selectivity to CH_4_ relative to those on the surface of SiXNS, and this catalytic performance can be further improved under photothermal reaction conditions. With evidence from in-situ infrared studies, two distinct pathways for CO_2_ methanation are observed for these two classes of materials, providing the insight and designing strategy for composite catalysts and materials with structural confinement effects.

## Results

### Materials design and fabrication process

**Fig. 1:**
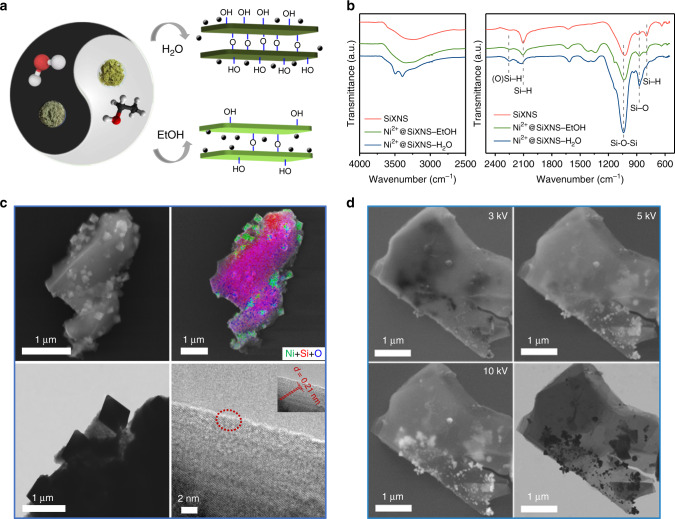
Preparation, growth, and electron microscopy characterization of NiO@SiXNS. **a** Structures of two different Ni^2+^-impregnated SiXNS samples. The taijitu symbolizes the fact that, when used as the solvent, H_2_O and EtOH, result in two distinct composite structures, despite being derived from the same solid siloxene precursor. The black spheres around the green SiXNS slabs indicate the Ni^2+^ ions. **b** FTIR spectra of pristine SiXNS and SiXNS impregnated with Ni^2+^ ions in EtOH and H_2_O. **c** Scanning electron microscopy (SEM) with energy-dispersive X-ray (EDX) and transmission electron microscopy (TEM) images of NiO@SiXNS-H_2_O, and **d** SEM and TEM images of NiO@SiXNS-EtOH

SiXNS were exfoliated from bulk calcium silicide at 0 °C using concentrated HCl^27,32,33^. The composition and structure of such exfoliated 2D nanosheets have been studied for decades. Three main types of structures containing Si–H and different types of O sites have been proposed: first, the Weiss siloxene consisting of corrugated Si (111) layers terminated above and below by OH and H groups^34^; second, the Kautsky siloxene consisting of puckered Si six rings interconnected by oxygen bridges^35^; and third, an unusual structure wherein linear Si chains are interconnected via oxygen bridges and terminated by hydrogen^27^. When actually synthesized in the lab, a mixture of the above mentioned structures with the general formula of Si_6_O_*x*_(OH)_*y*_H_6-*y*_ is observed, in which Si is terminated by –H and –OH functional groups, with Si–O–Si bridges also existing^36,37^. Such configurations allow for dispersion in common polar solvents, such as water and ethanol, facilitating the next step of nanocomposite formation using the soluble Ni salt. A dispersion of the fresh SiXNS was mixed and sonicated with a nickel nitrate solution, dried by heating under vacuum, and then calcined under air at 400 °C for 3 h. This procedure resembles the widely used impregnation method for producing conventional supported metal catalysts; however, it was found that the choice of solvent (i.e., water or ethanol), resulted in different structures in the product material. Figure 1a illustrates the philosophy of the design strategy: the circle of Taiji represents the unity of beginning from the same precursor, whereas the separated black and white halves symbolize the difference of the final products. When using water as the solvent, both the hydrolysis of the Si–H perpendicular to the plane to form Si–OH and the condensation of Si–OH into Si–O–Si will proceed during the impregnation process, as was also observed by Weiss et al.^34^ (the pioneer of the siloxene material) and, more recently, by Xie et al.^27^. This hydrolysis by water appears to inhibit the infiltration and deposition of nickel nitrate between the siloxene sheets. In contrast, ethanol preserves more Si–H and results in less condensation of Si–OH. These mechanisms are evidenced by Fourier-transform infrared (FTIR) spectroscopy (Fig. 1b) of samples prepared using either water or ethanol. The spectrum of the SiXNS impregnated in ethanol resembles that of the original pristine SiXNS, with the extra peaks at around 1350 cm^−1^ assigned to nitrate ions. In contrast, the sample impregnated in water exhibits significantly increased Si–O–Si peaks due to condensation of Si–OH, a weak mode at around 2100 cm^−1^ for Si–H, and distinctive Si–OH modes between 3250 and 3500 cm^−1^, due to more extensive hydrolysis of Si–H. As a result, following the decomposition of nickel nitrate via calcination, the NiO so produced tended to be located on the more extensively hydrolyzed exterior surface of the stacked SiXNS impregnated in water (NiO@SiXNS-H_2_O) but between the sheets of less-hydrolyzed SiXNS impregnated in ethanol (NiO@SiXNS-EtOH). Due to this unique siloxene chemistry, significantly different final structures were obtained from the same precursor material. Notably, the Ni loadings were almost the same for NiO@SiXNS-H_2_O and NiO@SiXNS-EtOH, (4.9 and 4.7 wt%, respectively), as determined using inductively coupled-plasma atomic emission spectroscopy (ICP-AES).

The morphology and microstructure of fresh NiO@SiXNS was examined using electron microscopy; representative images are shown in Fig. 1c, d. Scanning electron microscopy (SEM) and transmission electron microscopy (TEM) images of NiO@SiXNS-H_2_O showed that NiO nanoparticles were deposited on the external surface of SiXNS. This can be further illustrated by the patterns of green (Ni) octahedra scattered across the red (Si) support, whereas the blue (O) component is more homogeneously distributed throughout the material, as obtained from energy-dispersive X-ray (EDX) mapping analysis (Fig. 1c). The lattice fringe of 0.21 nm on NiO@SiXNS-H_2_O originates from the NiO (200) plane, further confirming the identity of the phase. In contrast, NiO nanoparticles formed on NiO@SiXNS-EtOH grew between the sheets of SiXNS. In the SEM images shown in Fig. 1d, very few particles could be observed on the exterior surface. Only by increasing the accelerating voltage could more and brighter particles be revealed, exhibiting distinctive contrast against the surrounding SiXNS as the penetration ability of the accelerated electrons increased. Providing crucial support for this observation is the fact that TEM imaging clearly revealed the majority of NiO particles were encapsulated within the SiXNS and could not be observed using low-voltage SEM.

### Characterization of NiO@SiXNS materials

**Fig. 2:**
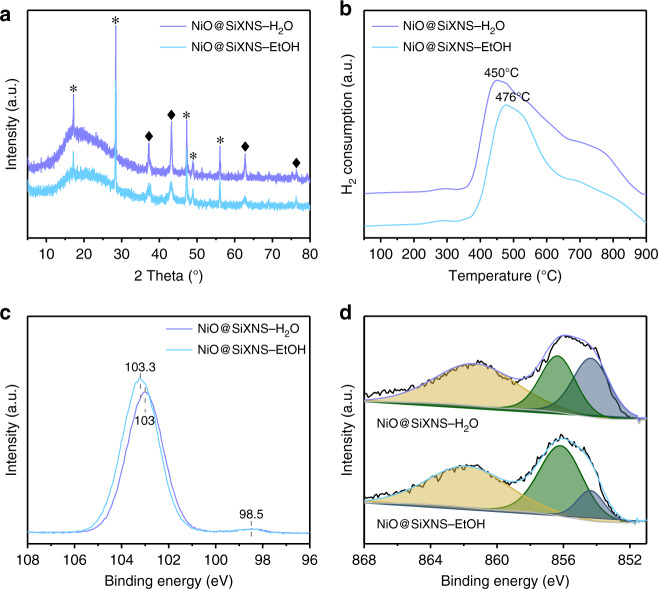
Characterization of NiO@SiXNS materials. **a** PXRD patterns showing characteristic peaks from the (*) SiXNS and (♦) NiO constituents of the composite materials. **b** H_2_–TPR (hydrogen temperature programmed reduction) plots indicating the appreciable difference in the onset of NiO reduction on heating under a hydrogen atmosphere. **c**, **d** XPS spectra illustrating the differences in the local bonding environments of **c** Si 2*p* and **d** Ni 2*p*_3/2_ obtained from of NiO@SiXNS samples (green peak refers to NiSiO_3_, cyan peak corresponds to NiO, and yellow one is the satellite peak)

Powder X-ray diffraction (PXRD) (Fig. 2a) studies of the NiO@SiXNS-H_2_O and NiO@SiXNS-EtOH were carried out in order to better understand their structure. In addition to the peaks associated with SiXNS exfoliated from CaSi_2_ (marked with asterisks)^37^, both samples exhibited one broad peak at ~ 20° (corresponding to the presence of an amorphous phase) and four obvious peaks (marked with rhombuses) corresponding to NiO. The slightly broader peaks of NiO in NiO@SiXNS-EtOH also implies that spatial confinement limits the nucleation and growth process. The Brunauer–Emmett–Teller (BET) surface areas of NiO@SiXNS-H_2_O and NiO@SiXNS-EtOH are 8.8 and 9.1 m^2^ g^−1^, respectively (Supplementary Fig. 1).

The reduction properties of NiO@SiXNS was measured by using hydrogen temperature programmed reduction (Fig. 2b). Slight hydrogen consumption begins at 250 °C for both samples. A major peak was observed at 450 °C for NiO@SiXNS-H_2_O, whereas the corresponding peak was not observed until 476 °C for NiO@SiXNS-EtOH, which also exhibited a shoulder peak at ~ 700 °C. The major peak in these TPR profiles originates from the reduction of NiO. The higher reduction temperature of NiO@SiXNS-EtOH relative to NiO@SiXNS-H_2_O is attributed to the fact that, in the former case, NiO nanoparticles are trapped inside the SiXNS and well-anchored to the support, whereas, in the latter, NiO nanoparticles are mainly located on the exterior where they are more exposed to the hydrogen reductant. The small peak at ~ 700 °C on the TPR profiles of NiO@SiXNS is attributed to the presence of silicate species^38^.

XPS spectroscopy (Fig. 2c, d) revealed more detailed information about NiO@SiXNS. The Si 2*p* spectra showed key peaks at the binding energies of 98.5 and 103 eV, corresponding to siloxene Si^0^ and Si^4+^ sites, respectively. The predominance of Si^4+^ compared with Si^0^ can be attributed to oxidation during the calcination process. In addition, spectra from both samples exhibited broad, asymmetric peaks in the Ni 2*p*_3/2_ region. This region consisted of two peaks at binding energies of 854.5 and 856.3 eV, which represent NiO and a nickel silicate (NiSiO_3_) phase, respectively^39^. According to the deconvoluted XPS results, the NiSiO_3_-to-NiO peak area ratio of NiO@SiXNS-EtOH (3.6) was four times of that for NiO@SiXNS-H_2_O (0.9). This difference further supports the preferred location of NiO at interior sites in NiO@SiXNS-EtOH, as more NiO should be sandwiched between SiXNS.

### Characterization of Ni@SiXNS materials

**Fig. 3:**
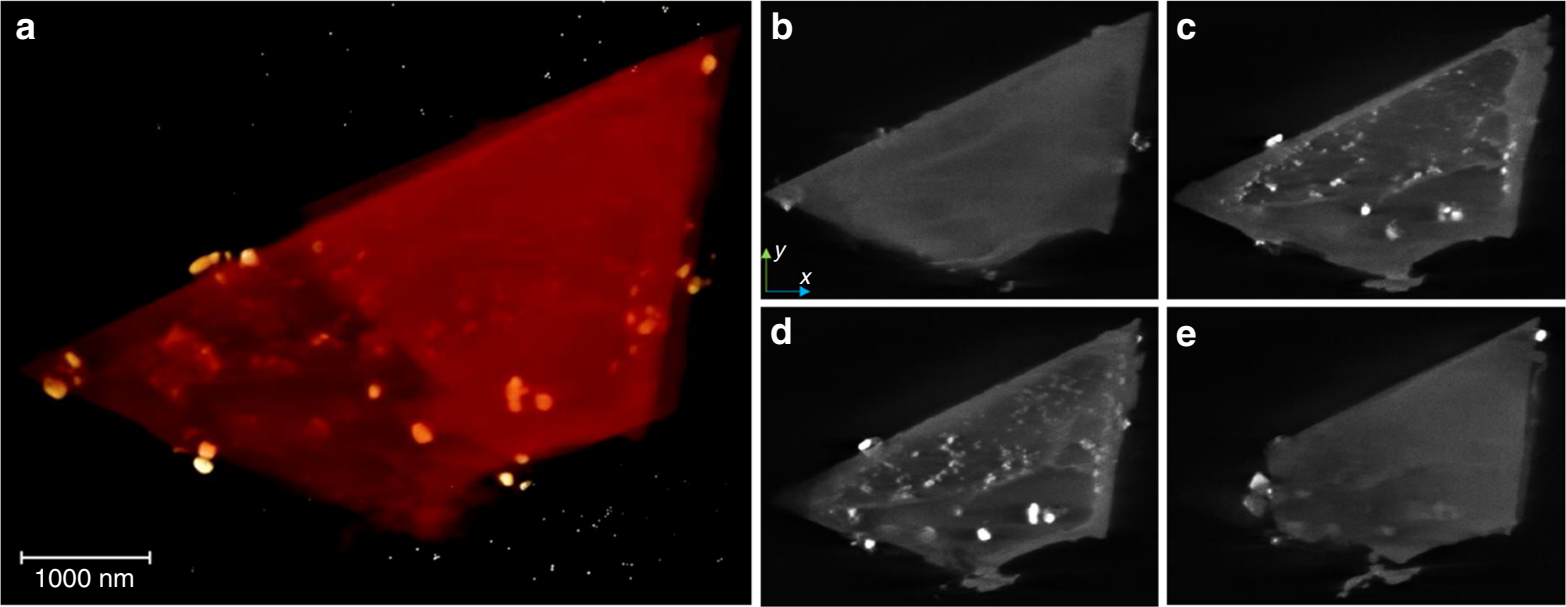
Electron tomography analysis of Ni@SiXNS-EtOH. **a** A screenshot image depicting the volume rendering of the 3D reconstruction and **b**–**e** images of slices obtained at different *z* depths of the 3D reconstruction of a particulate of Ni@SiXNS-EtOH. Slices shown in **b** and **e** reveal almost no Ni-containing nanoparticles on the top and bottom of the composite particulate, whereas those shown in **c**, **d** reveal the presence of Ni-containing nanoparticles in the interlayer spaces between SiXNS. Tiny, scattered white dots in the background of **a** are the added gold nanoparticles as track markers

In order to determine the structure of the catalysts, the reduced Ni@SiXNS were further characterized. Accurate 3D visualization of the confined architecture of Ni@SiXNS-EtOH was achieved via electron tomography analysis (Fig. 3 and Supplementary Movie 1). The majority of the nanoparticles are seemingly encapsulated within the interlayer spaces of adjacent nanosheets (Fig. 3a and Supplementary Movie 1). Almost no nanoparticles were observed on the top and bottom of the nanosheets (Fig. 3b, e); in contrast, most of the nanoparticles with bright contrast were revealed in slices deeper within the 3D reconstruction of this composite particulate (Fig. 3c, d).

**Fig. 4:**
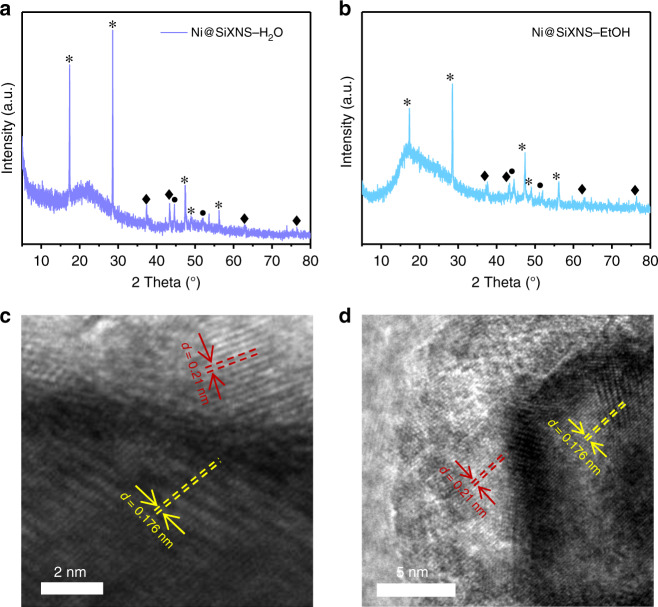
Characterization of Ni@SiXNS Materials. **a**, **b** PXRD patterns showing characteristic peaks from the (●) Ni, (♦) NiO, and (*) SiXNS constituents of **a** Ni@SiXNS-H_2_O and **b** Ni@SiXNS-EtOH. **c**, **d** TEM images of **c** Ni@SiXNS-H_2_O and **d** Ni@SiXNS-EtOH

The PXRD patterns of Ni@SiXNS catalysts revealed that both Ni and NiO were present following reduction (Fig. 4a, b). TEM images of the Ni@SiXNS-H_2_O and Ni@SiXNS-EtOH, shown in Fig. 4c and Supplementary Fig. 2, further revealed that Ni nanoparticles were exposed on the exterior of the SiXNS, and that well-defined lattice fringes of 0.176 and 0.21 nm associated with Ni (200) and NiO (200) planes are observed on Ni@SiXNS-H_2_O. On Ni@SiXNS-EtOH, the majority of Ni nanoparticles are sandwiched between SiXNS sheets and only a few Ni nanoparticles are immobilized on the outer surface as seen in the thinner regions of SiXNS (Supplementary Fig. 3). The lattice fringes of 0.176 nm and 0.210 nm can also be ascribed to Ni (200) and NiO (200) planes, respectively (Fig. 4d). The Ni surface area and size of Ni@SiXNS were determined using H_2_ chemisorption measurements. Similar Ni surface areas of 3.8 and 4.0 m^2^ g_cat_^−1^ were observed for the Ni@SiXNS-H_2_O and Ni@SiXNS-EtOH, respectively. The sizes of Ni particles were also similar, being 10.7 nm for Ni@SiXNS-H_2_O and 10.3 nm for Ni@SiXNS-EtOH. These resemblances suggest that the surface area and size of Ni in these catalysts are not responsible for the observed differences in their catalytic performance.

### Catalytic performance of CO_2_ methanation

**Fig. 5:**
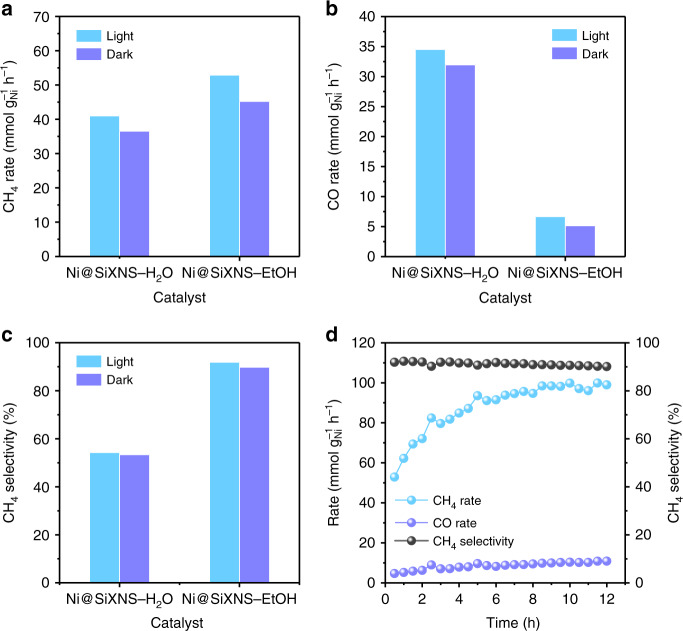
Catalytic performance of CO_2_ methanation on Ni@SiXNS. **a**, **b** Observed catalytic rates for **a** CH_4_ production and **b** CO production by Ni@SiXNS samples under light and dark conditions. **c** CH_4_ selectivity of Ni@SiXNS samples for CO_2_ methanation under light and dark conditions. **d** CH_4_ production rate and selectivity as well as CO production rate of Ni@SiXNS-EtOH for CO_2_ methanation under illumination for a continuous 12 h at atmospheric pressure

The catalytic activity of the Ni@SiXNS catalysts for the CO_2_ methanation was tested at 300 °C in a flow reactor system operating at atmospheric pressure under a flowing H_2_/CO_2_ atmosphere (4:1). The pristine SiXNS (calcined at 400 °C for 3 h) showed no activity toward CO_2_ methanation either in the dark or light under the same reaction conditions. For the composite catalyst materials, however, significant activity was observed, with CH_4_ and CO being identified as the only products generated by these Ni@SiXNS catalysts under both dark and light conditions (Fig. 5). The CH_4_ and CO production rates for Ni@SiXNS-H_2_O in the dark were found to be 36.6 and 32.0 mmol g_Ni_^−1^ h^−1^, respectively, which increased to 41.0 and 34.5 mmol g_Ni_^−1^ h^−1^ on exposure to light (Fig. 5a, b). Despite this rate increase, CH_4_ selectivity remained virtually unchanged, with calculated values of 53.4% and 54.3% upon switching from dark to light operating conditions (Fig. 5c). This slightly enhanced performance is attributed to the photothermal effect of the Ni particles in Ni@SiXNS-H_2_O. The diffuse reflectance spectrum of the sample is low throughout the visible spectral region because of the significant visible light-harvesting capacity (interband and intraband electronic transitions) of the black Ni nanoparticles, which should favor local heating of Ni via the photothermal effect (Supplementary Fig. 4)^40^.

As a control experiment, commercial amorphous silicon dioxide-supported nickel (Ni@SiO_2_) was employed and loaded into the same flow catalyst reactor for evaluation under the same conditions. The Ni loading and mass of the Ni@SiO_2_ used were the same as those of Ni@SiXNS. As shown in Supplementary Fig. 5, the CH_4_ and CO production rates in the dark over Ni@SiO_2_ are 22.78 and 193.70 mmol g_Ni_^−1^ h^−1^, respectively. In this case, CH_4_ selectivity is only 10.5%, significantly lower than that of Ni@SiXNS-H_2_O. Under light conditions, CH_4_ and CO production rates increase to 34.55 and 296.09 mmol g_Ni_^−1^ h^−1^, whereas CH_4_ selectivity still remains at 10.5%. The result indicates that Ni supported on SiO_2_ favors the reverse water–gas shift reaction, contributing to the formation of large amount of CO rather than CH_4_. We attribute the lower selectivity toward CH_4_ to the poorer contact between Ni and commercial spherically shaped SiO_2_ particles compared with that between the Ni and the planar SiXNS.

In an attempt to improve the selectivity toward the production of CH_4_, Ni@SiXNS-EtOH with Ni nanoparticles trapped between the sheets was also tested for its CO_2_ methanation activity, as shown in Fig. 5. Notably, CH_4_ and CO production rates for this catalyst increased from 45.2 and 5.1 mmol g_Ni_^−1^ h^−1^, respectively, in the dark to 52.9 and 6.7 mmol g_Ni_^−1^ h^−1^ in the light (Fig. 5a, b). The CH_4_ selectivity increased slightly from 89.8% in the dark to 91.9% in the light (Fig. 5c). Isotopic labeling experiments using ^13^C and gas chromatography-mass spectrometry (GC-MS) were used to study the products generated from a ^13^CO_2_ reactant feed. The mass-to-charge (*m*/*z*) ratios observed at 17 and 29 amu in the GC-MS spectrum corresponded to ^13^CH_4_ and ^13^CO (Supplementary Fig. 6), confirming that CH_4_ and CO are, indeed, produced from the methanation of CO_2_ on Ni@SiXNS and not from adventitious carbon contaminates. Thus, Ni@SiXNS-EtOH exhibited both higher selectivity and activity for the CO_2_ methanation than Ni@SiXNS-H_2_O.

The catalytic stability at 300 °C with light of Ni@SiXNS-EtOH was next investigated, as illustrated in Fig. 5d. The CH_4_ production rate increased progressively and reached a stable value around an impressive 100 mmol g_Ni_^−1^ h^−1^ during the 12 h test, whereas the CO production rate increased only slightly and stabilized around 10 mmol g_Ni_^−1^ h^−1^. In addition, CH_4_ selectivity was observed to remain stable at above ~90%. One can therefore deduce that Ni@SiXNS-EtOH is a highly active, selective, and stable catalyst for the preferential hydrogenation of CO_2_ to CH_4_. The catalytic performance of an industrial Ni@Al_2_O_3_ catalyst is shown as a reference for comparison in Supplementary Fig. 7. CH_4_ and CO production rates in the light over Ni@Al_2_O_3_ are 28.69 and 0.63 mmol g_Ni_^−1^ h^−1^ with a CH_4_ selectivity of 97.8% in the first 30 min and slightly decreased and maintained at 27.78 and 0.55 mmol g_Ni_^−1^ h^−1^ with similar selectivity. Although Ni@Al_2_O_3_ has higher CH_4_ selectivity than that of Ni@SiXNS-EtOH, the production rates are much lower.

### Catalytic reaction pathways

To gain deeper insights into the CO_2_ methanation reaction over Ni@SiXNS, in-situ diffuse reflectance infrared Fourier transform spectroscopy (DRIFTS) studies were conducted. Each NiO@SiXNS sample was reduced in the DRIFTS sample cell for 1 h under a H_2_ atmosphere and then purged for 1 h under He at 300 °C. Next, the sample was exposed to a mixture of CO_2_ (2 s.c.c.m.), H_2_ (8 s.c.c.m.), and He (10 s.c.c.m.) for 1 h. We focused our analysis on the IR fingerprint regions of 1200–2200 cm^−1^ and 2600–3400 cm^−1^ in the consecutive spectra, where reaction intermediates could be detected.

**Fig. 6:**
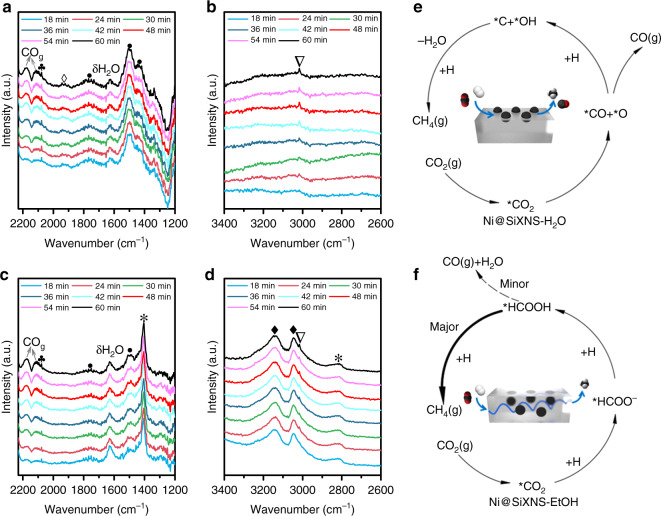
In-situ DRIFTS experiments and proposed catalytic pathways. **a**–**d** In-situ DRIFTS spectra of samples Ni@SiXNS-H_2_O (**a**, **b**) and Ni@SiXNS-EtOH (**c**, **d**). **e**, **f** The catalytic reaction pathways of Ni@SiXNS-H_2_O- (**e**) and Ni@SiXNS-EtOH- (**f**) catalyzed CO_2_ reduction and the key reactions involved that can be detected by DRIFTS. Symbols: (♣) linear CO; (◊) bridging CO; (●) carbonate; (∇) CH_4_; (*) formate; (♦) OH groups

For Ni@SiXNS-H_2_O, two key reaction intermediates were observed, as shown in Fig. 6a. The first, identified as chemisorbed CO_2_ had observable peaks at 1430 and 1500/1767 cm^−1^, and is diagnostic of the formation of bicarbonate and carbonate species, respectively^41–44^. The second was identified from peaks located at 1930 and 2077 cm^−1^, which are attributed to the presence of bridging and linearly adsorbed CO molecules on Ni, respectively^45,46^. As depicted in Fig. 6b, C–H stretching vibrations of CH_4_ were also detected at 3016 cm^−1^, with additional peaks at 2111/2174 and 1630 cm^−1^ being observed for gaseous CO and H_2_O, respectively^6^.

For Ni@SiXNS-EtOH, apart from the carbonate species, one important reaction intermediate appeared at 1404 cm^−1^ in the spectra (Fig. 6c) and was ascribed to a surface formate species (HCO_2_^−^)^47,48^; this assignment is supported by an additional peak observed at 2814 cm^−1^ (Fig. 6d)^11,49^. It is noteworthy that the peak intensity of this formate species is much higher than that of the carbonate species. Yet, another species with corresponding peaks at 3046 and 3140 cm^−1^ matched well with those of hydroxyl groups, as reported in the literature^50^. It should be noted that no obvious peaks could be detected in the range of 2000–1800 cm^−1^ for Ni@SiXNS-EtOH, which reveals the essential absence of bridging CO; this is likely because Ni nanoparticles in the catalyst are trapped between sheets of SiXNS, thus decreasing the chance for the bonding of CO molecules to multiple Ni atoms. Only gaseous CO appears in the spectra, as well as gaseous CH_4_ and H_2_O.

Based on the differential DRIFTS spectra of the Ni@SiXNS catalysts, a reaction pathway for CO_2_ methanation was proposed (Fig. 6e, f). As the Ni nanoparticles were located on the exterior surface of SiXNS in Ni@SiXNS-H_2_O, these particles are readily exposed to CO_2_, whereupon the chemisorbed *CO_2_ species can dissociate into *CO and *O. The strongly bound, bridge-bonded *CO species indicated by the IR data can further dissociate to carbon species (*C), which can then be hydrogenated to form CH_4_ and desorb from the catalyst surface, corresponding to the observed CH_4_ production, whereas the *O species can react with hydrogen to produce H_2_O. However, the stable bridging *CO species also have a higher chance to convert to CO as the final product, leading to an overall lower selectivity to CH_4_.

In contrast, Ni nanoparticles in Ni@SiXNS-EtOH are confined within SiXNS and the bridging *CO species cannot be readily formed. A recent theoretical calculation has shown that a silica-cluster-modified Ni (111) surface has a higher activation energy barrier for the dissociation of *CO_2_ to CO through the *CO pathway than a clean exposed Ni (111) surface^51^. Our DRIFTS spectra and catalytic performance tests on Ni@SiXNS-EtOH are consistent with the prediction that the generation of *CO, and thus CO, is limited due to the Ni nanoparticles being encased within the SiXNS. Therefore, the chemisorbed *CO_2_ species for this structure could instead convert first to HCO_2_^−^ and then to formic acid (HCOOH). More pronounced C–H IR mode intensity from HCOOH could be detected by quickly cooling down the reactor during DRIFTS measurements (Supplementary Fig. 8)^49^. A small portion of the HCOOH so generated could decompose to gaseous CO and H_2_O; however, a larger proportion is likely to participate in the rapid generation of CH_4_, possibly by the formate pathway reported by Kattel et al.^52^. This additional pathway for CH_4_ production on Ni@SiXNS-EtOH could be due to the tortuosity experienced by CO_2_ within the interior of the catalyst, which enhances the probability of it being fully hydrogenated to CH_4_ via the associative CO_2_ methanation mechanism (in which carbonate is hydrogenated to bicarbonate and then quickly dehydrated to formate)^53^. Besides, the newly generated OH groups observed in Fig. 6d could provide additional H source and aid in formate formation^47^.

## Discussion

The synthesis of Ni@SiXNS nanocomposites relies on a distinctive kind of silicon surface chemistry in which the Si–H hydrolysis and Si–OH condensation are selectively triggered or avoided. The resulting Ni@SiXNS materials are extremely active toward the conversion of CO_2_ to CH_4_ with high selectivity and impressive stability. The nucleation and growth of Ni nanoparticles on either the exterior or interior of the SiXNS was solvent-sensitive, enabled by choosing water or ethanol, respectively. After a standard impregnation–calcination–reduction in water, a Ni@SiXNS-H_2_O catalyst was produced. For this material, Ni nanoparticles were immobilized on the exterior surface of the SiXNS. The corresponding CH_4_ production rate was 41.0 mmol g_Ni_^−1^ h^−1^ with heating at 300 °C with assistance of light, but CH_4_ selectivity was only 54.3%. In contrast, the Ni@SiXNS-EtOH catalyst favored Ni located within the interlayer space of the SiXNS. These samples featured a CH_4_ production rate of 52.9 mmol g_Ni_^−1^ h^−1^ and a CH_4_ selectivity over 90%. This spatially confined Ni catalyst proved stable in the light and, over time, eventually reaching an impressive CH_4_ production rate of ~ 100 mmol g_Ni_^−1^ h^−1^ with ~90% selectivity.

An in-situ infrared study clarified the reaction pathways of the Ni@SiXNS catalysts for CO_2_ methanation, revealing the origin of the observed differences in selectivity between the two types of structures. Distinct reaction intermediates (i.e., CO and formate species) were observed on the Ni@SiXNS-H_2_O and Ni@SiXNS-EtOH samples, respectively. For Ni@SiXNS-H_2_O, the exposed Ni on the surface of SiXNS could easily bridge-bond to CO, leading to the hydrogenation of *C to CH_4_ following CO dissociation. For Ni@SiXNS-EtOH, the confined Ni sites are unable to form bridging *CO species and formate species were instead the main intermediates leading to CH_4_ production.

The gradual increase of the CH_4_ production rate on Ni@SiXNS-EtOH in Fig. 5d originated from the gradual release of Ni during the CO_2_ methanation reaction, corresponding well with the recent popular catalytic concept of the “sustained release catalyst,” designed to maintain the high reactivity and stability of the obtained catalyst^54,55^. A thorough in-situ Extended X-Ray Absorption Fine Structure (EXAFS) study illustrated the gradual evolution of Ni in Ni@SiXNS-EtOH under simulated reaction conditions (Supplementary Fig. 9). Due to the fact that the reaction under light conditions probably resulted in a higher reactor temperature than 300 °C, and that the high flow rate would bring down the surface temperature, we performed the in-situ EXAFS study at 330 °C at a flow of 4:1 H_2_/CO_2_ (i.e., 130 s.c.c.m. 3.5% H_2_ in He and 60 s.c.c.m. 2% CO_2_ in He) after H_2_ activation at 300 °C. Significant changes in the XAS spectra began to occur and continued over a 90 min period. The changes continued but gradually slowed and so the temperature was again increased to 350 °C in order to more rapidly drive the sample toward redox equilibrium, which was reached within the following 90 min period. This matched well with the rapid attainment of a steady CH_4_ or CO formation rate in a stability test of Ni@SiXNS-EtOH under dark conditions at 300 °C with pre-reduction at 350 °C (Supplementary Fig. 10).

On the origin of high selectivity for CH_4_ production of Ni@SiXNS-EtOH, apparent reaction orders were estimated from the variation in production rates with different inlet reactant concentrations according to the rate law equation *r* = *kP*_H2_^a^*P*_CO2_^b^ (Supplementary Fig. 11, Supplementary Table 1, and Supplementary Notes) after the sample was stabilized, as described above. The apparent reaction orders of CO_2_ and H_2_ were 0.40 and 1.56, respectively, for CH_4_ production, whereas the reaction orders of CO_2_ and H_2_ were 2.80 and 0.28 for CO production. Thus, the relatively large reaction order value of H_2_ implies dominant control of the CH_4_ production rate by the partial pressure of H_2_, which is indicative of the associative methanation pathway in which H_2_ fully hydrogenates the activated CO_2_. In contrast, for CO production, the much larger reaction order of CO_2_ indicates its greater dependence on CO_2_ adsorption and dissociation.

Over the range of H_2_ partial pressure variations explored, it was determined that the fractional yield of CH_4_ from CO_2_ was consistently larger than that for CO production from CO_2_, indicating high selectivity toward CH_4_ production. Further analysis revealed the rate-limiting step to be the decomposition and further hydrogenation of formate-type species HCOOH* (see Supplementary Information).

The crucial finding of this study is that control over the location of the Ni nanoparticles, either at the interior or exterior regions of the SiXNS, matters when it comes to selectivity favoring the methanation vs. the reverse water–gas shift reaction. The CO pathway dominates for Ni at the exterior of the nanosheets, whereas the formate pathway is preferred for Ni confined to the interior. Although the former does not discriminate much between CO and CH_4_ products, the latter strongly favors CH_4_. This innovative approach to the construction of designed architecture 2D Ni@SiXNS nanocomposites presents a paradigm in the field of gas-phase heterogeneous (photo)catalytic hydrogenation of CO_2_ to synthetic chemicals and fuels.

## Methods

### Synthesis of SiXNS and NiO@SiXNS

In a typical synthesis of SiXNS, 1.0 g of calcium silicide powder (Fluka™) was dispersed in 100 mL hydrochloric acid (36%, Caledon Laboratory Chemicals) pre-cooled to 0 °C in a jacketed flask with a circulator (NESLAB™ RTE-111). The mixture was stirred under inert atmosphere for 6 days at 0 °C. The solid product was collected via vacuum filtration, washed with anhydrous acetone, and dried under N_2_ flow before being stored under vacuum in a desiccator. For the synthesis of NiO@SiXNS-EtOH, 0.0124 g of nickel nitrate hexahydrate (Alfa Aesar) was dissolved into 1 mL of ethanol. Next, 0.048 g of SiXNS was dispersed into 2 mL of ethanol and sonicated for 30 min. Then the nickel nitrate ethanol solution was added into the SiXNS dispersion and sonicated for another 30 min. Next, it was placed at room temperature for 12 h and dried in vacuum at 70 °C for 24 h before being cooled down to room temperature under vacuum for 12 h. The powder sample was finally calcined at 400 °C in air for 3 h. The process for the synthesis of NiO@SiXNS-H_2_O was similar to that of NiO@SiXNS-EtOH, except that the solution was changed to an equal volume of distilled water.

### Synthesis of control samples

For NiO@SiO_2_, 0.0124 g of nickel nitrate hexahydrate (Alfa Aesar) was dissolved into 1 mL of distilled water. SiO_2_ (0.048 g; Aldrich, fumed, powder, 0.007 μm) was dispersed into 2 mL of distilled water and sonicated for 30 min. The nickel nitrate solution was then added into the SiO_2_ dispersion and sonicated for another 30 min. After that, it was held at room temperature for 12 h and dried in vacuum at 70 °C for 24 h. The powder sample was finally calcined at 400 °C in air for 3 h. The industrial Ni@Al_2_O_3_ catalyst was purchased from Riogen (10 wt% Ni) and used as received.

### Material characterizations

An ICP-AES (Thermo iCAP 6300 spectrometer) was used to measure the nickel content in the NiO@SiXNS samples. PXRD was performed on a Bruker D2 Phaser X-ray diffractometer, using Cu *K*_α_ radiation and an accelerating voltage of 30 kV. Nitrogen adsorption experiments were carried out at 77 K on a Quantachome Autosorb-1-C instrument. Prior to each adsorption measurement, the samples were degassed at 150 °C overnight under vacuum. The specific surface area was determined using the BET equation, applied to the best linear fit within the range of 0.05 ≤ *P*/*P*_0_ ≤ 0.35. FTIR was conducted by using a Perkin Elmer Spectrum One FTIR fitted with a universal attenuated total reflectance sampling accessory with a diamond-coated zinc selenide window. Diffuse reflectance of the samples was measured using a Lambda 1050 UV/Vis/NIR spectrometer from Perkin Elmer and an integrating sphere with a diameter of 150 mm. SEM and TEM images were taken on a Hitachi S-5200 high resolution “in lens” scanning electron microscope. EDX mapping was performed using an Oxford Inca EDX system. High-resolution TEM images of the samples were obtained on a FEI Tecnai G^2^-F20 system operated at 200 kV. For the electron tomography analysis, the powder of the Ni@SiXNS sample was dispersed on 100 × 400 mesh carbon-coated copper grids (Quantifoil), where 15 nm colloidal Au particles had been deposited before. Electron tomography was performed using a Fischione 2020 tomography holder on a FEI Titan 80–300 microscope operated at an acceleration voltage of 300 kV in scanning transmission electron microscopy (STEM) mode. High-angle annular dark field (HAADF) STEM tilt-series images were collected using the Xplore3D software (FEI) over the tilt range of ±76º with the increment of 2°. Alignment of the tilt series was performed using IMOD using the Au particles as fiducial markers and achieved a mean residual alignment error of 0.60 pixels. The aligned tilt series was reconstructed using the simultaneous iterative reconstruction algorithm within Inspect3D (FEI). The resultant tomogram had a final voxel size of 3.69 nm. The 3D image analysis and visualization of the reconstruction tomogram was performed in Amira 6.0. XPS was performed in an ultra-high vacuum chamber with base pressure of 10^−9^ Torr. The system used a Thermo Scientific K-Alpha XPS spectrometer. Samples were prepared for XPS analysis by adhering powders to carbon substrates; all peaks were energy-calibrated relative to the C1s peak of adventitious carbon at 284.8 eV and data analysis was carried out using CasaXPS software. The temperature-programmed reduction by H_2_ (H_2_-TPR) was measured on a chemisorption apparatus (Micromeritics AutoChem II 2920). In a typical experiment, 0.1 g of sample was pretreated at 300 °C for 1 h in He (30 s.c.c.m.). Afterwards, the system was cooled to 50 °C, followed by heating to 900 °C at a rate of 10 °C min^−1^ in 10% H_2_/He (30 s.c.c.m.), and the thermal conductivity detector (TCD) signal was recorded. The same apparatus was applied to perform H_2_ pulse chemisorption. The sample was first reduced at 300 °C for 1 h in 10% H_2_/He (30 s.c.c.m.), followed by cooling down to 35 °C in He (30 s.c.c.m.). A 10% H_2_/He gas composition was injected into the reduced sample at 5 min intervals, according to the pulse method, until saturation adsorption was observed. XAS spectra were recorded at Sector 9-BM of the Advanced Photon Source at Argonne National Laboratory (Lemont, IL). A Quick-EXAFS, double-crystal silicon monochromator was used to obtain time-resolved spectra, with each scan taking <30 s to complete. Gas ionization chambers were used to measure incident and transmitted X-ray intensities, and a Ni foil reference was used for in-line energy calibration of the system. A gas manifold consisting of mass flow controllers and Swagelok compression fittings was used to deliver reactant gases to the sample chamber.

Prior to analysis, a predetermined quantity of each sample was mulled together with a boron nitride bulking agent before being pressed into a solid pellet using an aluminum die and pistons. This die, still containing the pressed pellet, was then transferred into a sample chamber equipped with ceramic heating elements (top and bottom), a proportional-integral-derivative (PID) temperature controller, and an outer cooling jacket to prevent the Kapton® windows from being damaged at elevated temperatures. Processing of X-ray absorption near-edge structure (XANES) and EXAFS spectra was performed using Athena, part of the Demeter software package. The reaction chamber was first sealed and purged for several minutes with He gas at a flow rate of 190 s.c.c.m., following which the temperature was increased to 300 °C for reduction in a 190 s.c.c.m. flow of 3.5% H_2_ in He.

### Gas-phase CO_2_ reduction tests

Gas-phase photocatalytic reaction was conducted in a plug-flow capillary reactor in accordance with the procedure reported in our previous work^56^. For the catalytic investigation, the sample (~10 mg) was first heated to 300 °C in H_2_ (4 s.c.c.m.) and reduced in the dark for 1 h (yielding Ni@SiXNS). Next, a continuous flow of CO_2_ and H_2_ was introduced with a ratio of 1:4 (1 and 4 s.c.c.m., respectively) controlled by Alicat Scientific digital flow controllers. The catalytic performance of the samples was studied under both dark and light conditions, while being maintained at 300 °C for 30 min for each condition. The amounts of CO and CH_4_ produced were determined using online GC with autosampling (GC, Agilent 7820A, HP-PLOT Q PT, 19091P-QO3PT, 15 m*.320 mm*20 μm) directly connected to the reactor outlet and rates were calculated according to the calibration with standard gases. The stability measurement of the catalyst in photocatalytic reaction was investigated at 300 °C under light irradiation. In order to estimate the apparent reaction orders for CH_4_ and CO, the reactivity was stabilized by pre-treating the sample with H_2_ at a slightly higher temperature of 350 °C for 1 h and subsequently exposing it to CO_2_ and H_2_ at 300 °C for 10 h (Supplementary Fig. 10). A stable reactivity was almost reached within 2 h. In order to further confirm the production of CH_4_, isotopic labeling experiments were performed using ^13^CO_2_ (99.9 at%, Sigma‐Aldrich). Isotope distributions in the product gases were measured using an Agilent 7890A gas chromatograph-mass spectrometer with a 60 m GS-CarbonPLOT column leading to the mass spectrometer. For the rate law equation determination tests and thermal stability tests, CO_2_, H_2_, and Ar were flowed through in various ratios controlled by digital flow controllers (FMA-A2401, OMEGA). The concentration of CO and CH_4_ produced were determined using GC-MS (7890B and 5977A, He carrier, Agilent).

### In-situ DRIFT studies

In-situ DRIFTS experiments were performed on a Thermo Scientific™ Nicolet™ iS50 FT-IR Spectrometer with a mercury cadmium telluride detector cooled with liquid nitrogen. The spectrometer was equipped with a Harrick Praying Mantis™ diffuse reflection accessory and a Harrick high-temperature reaction chamber (HTC) with ZnSe windows. The temperature was controlled by a Harrick ATC-024-3 Temperature Controller. The spectrometer data were obtained using OMNIC software (Thermo Scientific). The autosampling function in the OMNIC software was used to collect data at specific time intervals. The loaded sample in the HTC was pressed carefully to ensure a flat and even surface. The following procedure for data collection was used. The sample was first reduced for 1 h at 300 °C in H_2_ (20 s.c.c.m.). Then the background scan was collected under flowing He at the same temperature, such that the spectra collected afterwards were differential spectra. Finally, CO_2_, H_2_, and He were introduced to the sample at a rate of 2, 8, and 10 s.c.c.m. The spectra were collected for 60 min with a resolution of 4 cm^−1^ and 32 scans.

## Supplementary information

Supplementary Information

Description of Additional Supplementary Information

Supplementary Movie 1

## Data Availability

The authors declare that the main data to support the finds of this study are available within the article and its Supplementary Information. The source data underlying all the plots in the main text and Supplementary Information are provided and available from the corresponding author upon request.

## Source data

Source Data
