# Supplementary material for: Nickel@Siloxene catalytic nanosheets for high-performance CO2 methanation
Source: Nat Commun. 2019 Jun 13;10:2608. doi: 10.1038/s41467-019-10464-x (PMC6565710; doi:10.1038/s41467-019-10464-x)
Supplement: Supplementary file 1 — Supplementary Information [file 41467_2019_10464_MOESM1_ESM.pdf]

Supplementary Information

Nickel@Siloxene Catalytic Nanosheets for High-Performance CO_2 Methanation

Xiaoliang Yan, Geoffrey A. Ozin

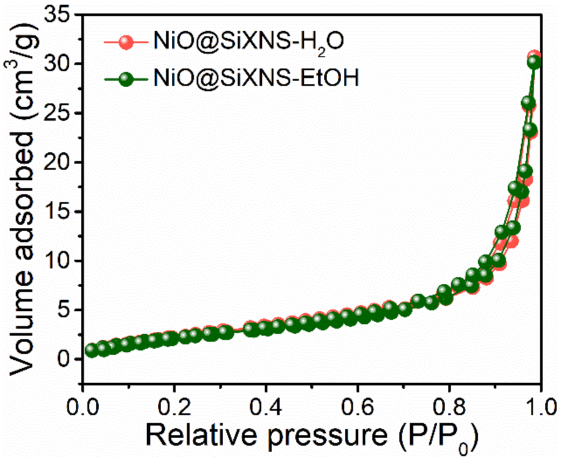

Supplementary Fig. 1. N_2 adsorption-desorption type III isotherms for NiO@SiXNS samples.

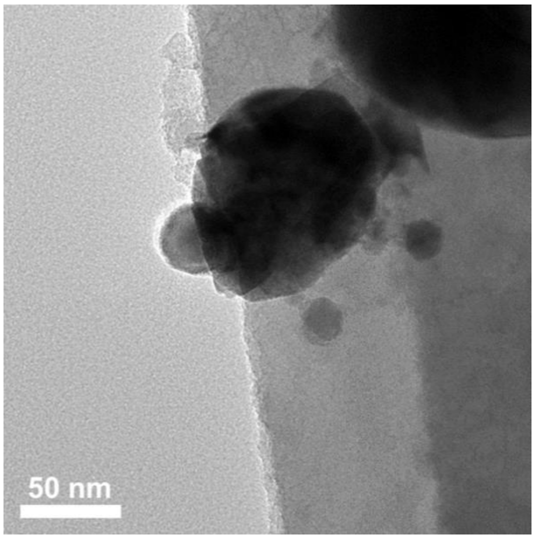

Supplementary Fig. 2. Representative TEM image of Ni@SiXNS-H_2O.

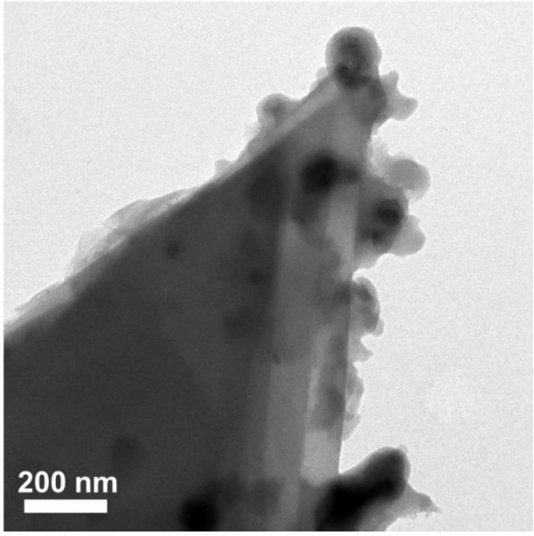

Supplementary Fig. 3. TEM image of Ni@SiXNS-EtOH captured at the edge of the composite material.

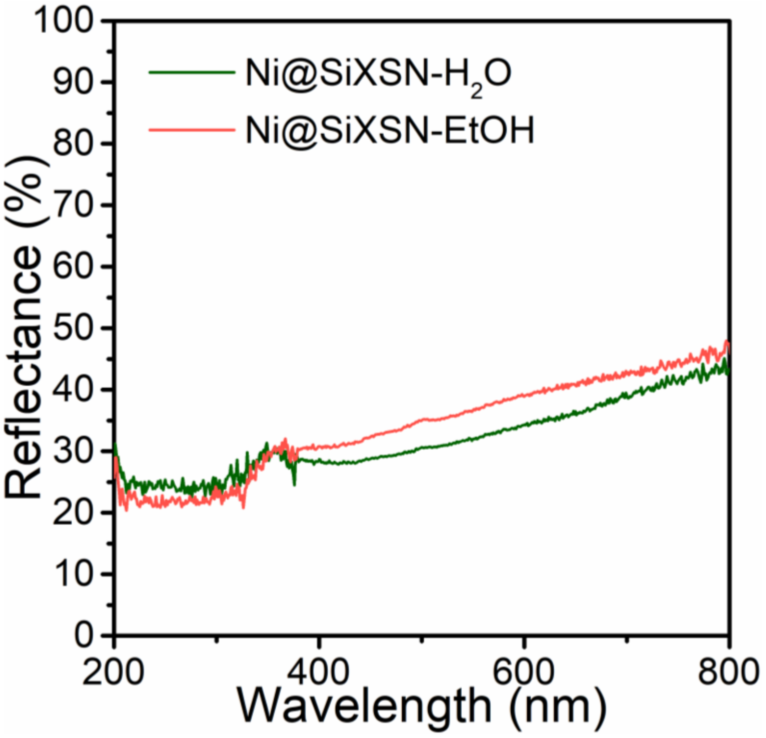

Supplementary Fig. 4. UV-vis diffuse reflectance spectra of Ni@SiXNS-H_2O and Ni@SiXNS-EtOH.

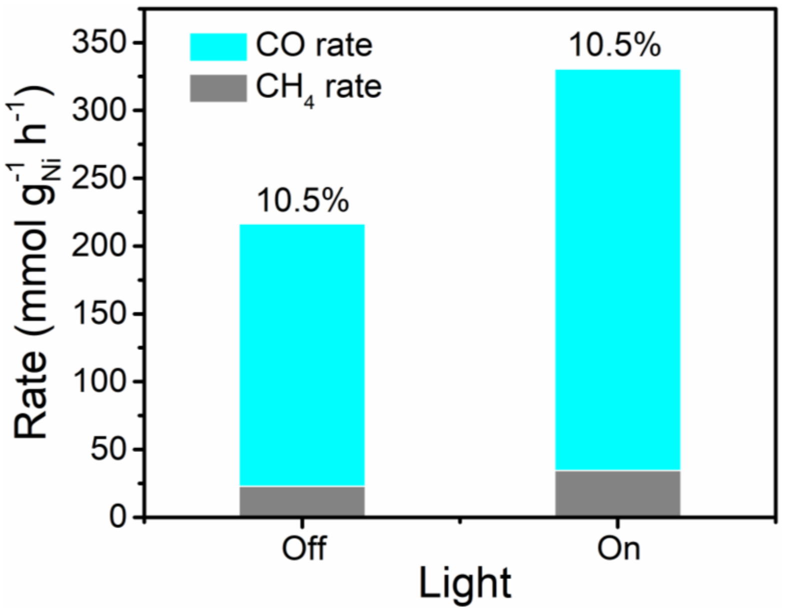

Supplementary Fig. 5. The performance of hydrogenation of CO_2 to methane on Ni@SiO_2.

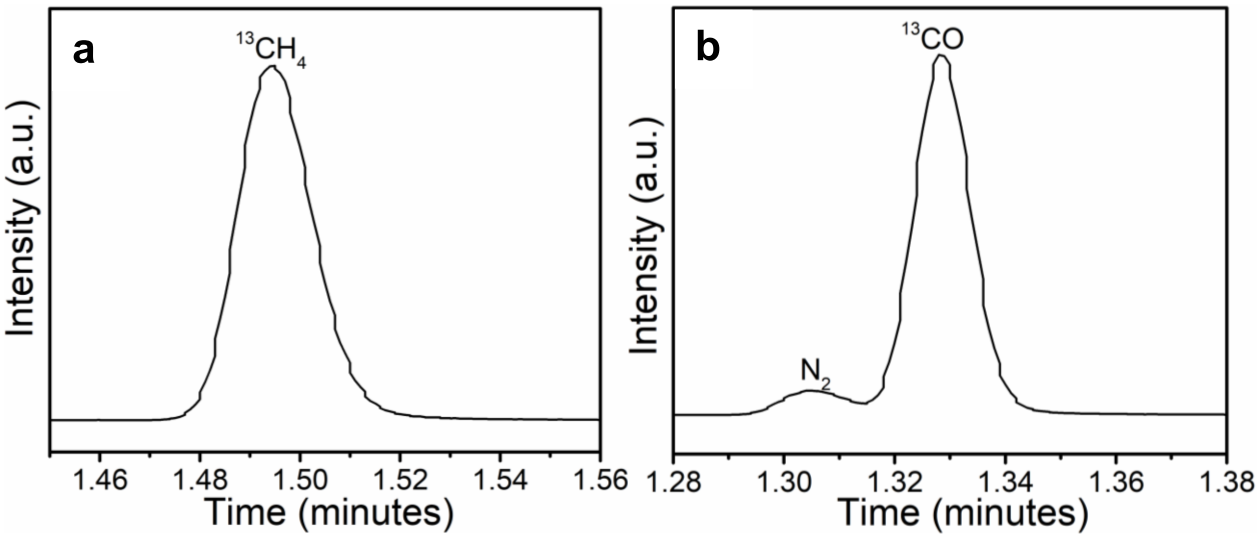

Supplementary Fig. 6. Mass spectra showing the ion mass (a) 17 and (b) 29 peaks for the $^{13}CO_2$ methanation in light over Ni@SiXNS-EtOH, confirming the products are from conversion of $^{13}CO_2$ input.

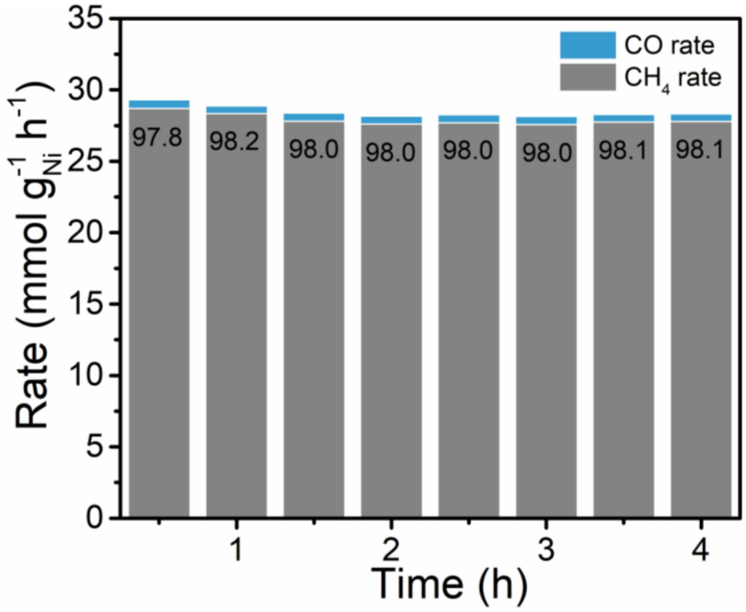

Supplementary Fig. 7. Catalyst test results for the hydrogenation of CO_2 on commercial 10 wt% Ni@Al_2O_3.

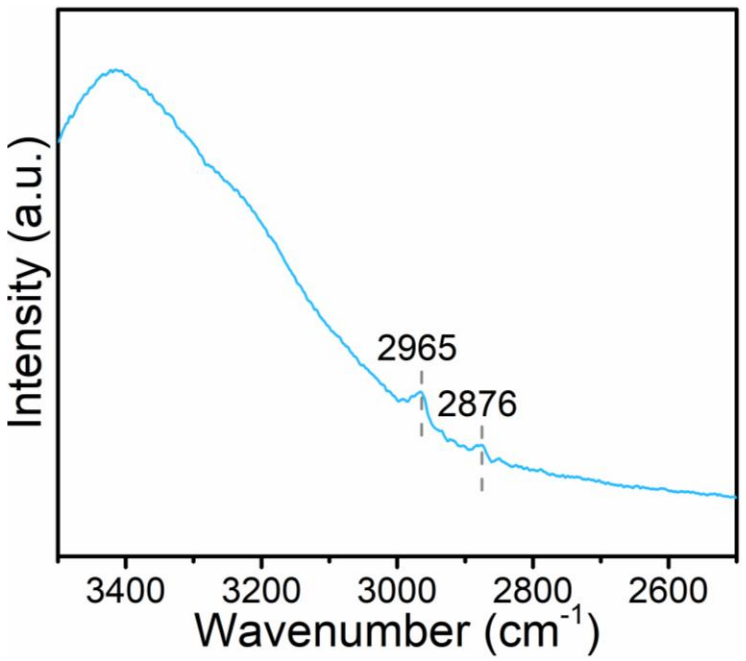

Supplementary Fig. 8. DRIFTS spectrum of Ni@SiXNS-EtOH showing two discernible peaks of C-H mode in HCOOH, acquired after performing *in-situ* CO_2 methanation for 60 min, followed by rapid cooling to 30 °C.

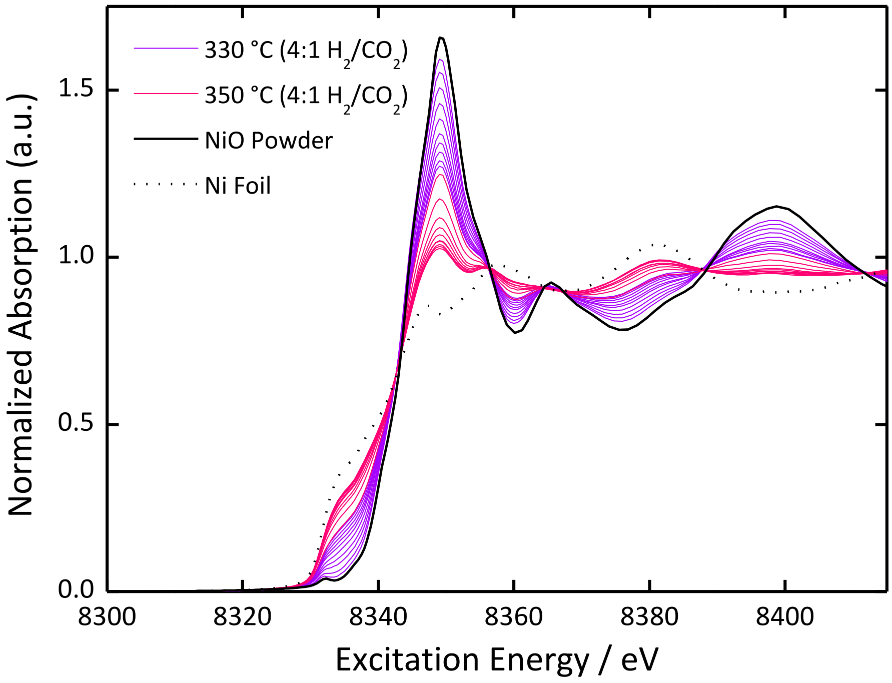

Supplementary Fig. 9. EXAFS spectra acquired at the Ni K-edge of the NiO@SiXNS-EtOH sample at different reaction temperatures with Ni foil and NiO powder reference.

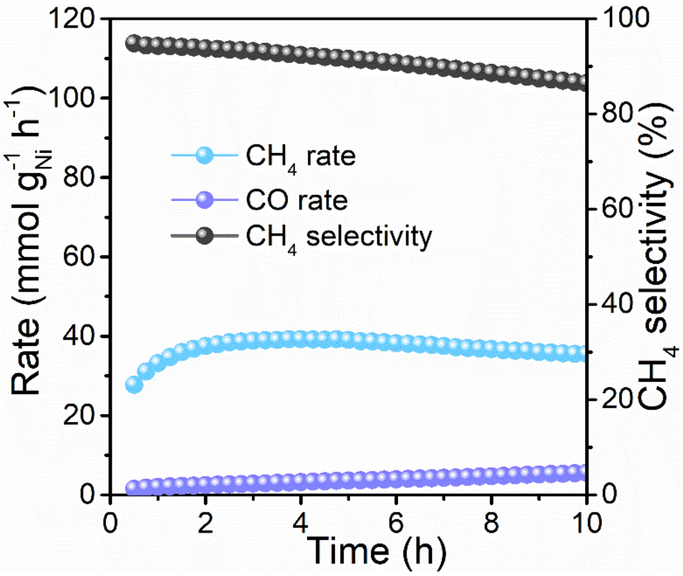

Supplementary Fig. 10. Rapid stabilization of CO_2 methanation by pre-treating the sample with H_2 at a slightly higher temperature of 350 °C and subsequently exposing it to CO_2 and H_2 at 300 °C for 10 h.

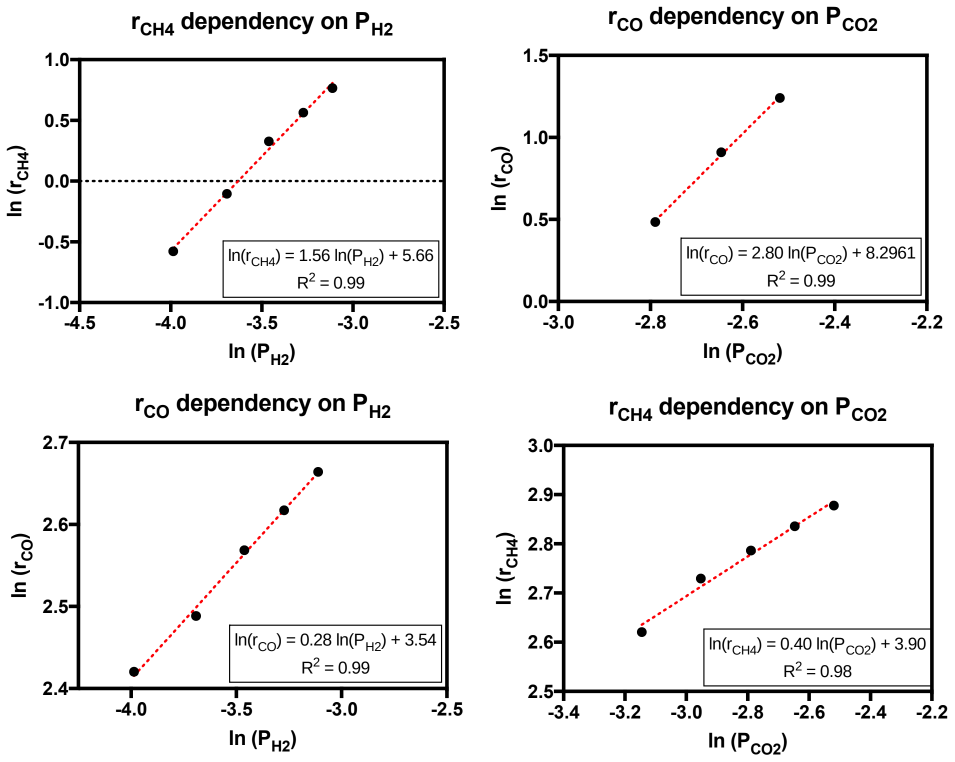

Supplementary Fig. 11. Derived from the rate law $r = kP_{H2}^{a}P_{CO2}^{b}$, the function $\ln(r) = \ln(k) + a\ln(P_{H2}) + b\ln(P_{CO2})$ is plotted to determine reaction orders versus partial pressures of H_2 and CO_2, keeping one fixed and the other varied.

Supplementary Notes

Rate Law Analysis:

Stoichiometric analysis of the parallel reaction network for CO_2 conversion to either CO or CH_4 provided the following relationships between the species in the system that were measured (CO and CH_4), and those that were not directly measured: $r_{CO_2} = r_{CO} + r_{CH_4}$, $r_{H_2} = r_{CO} + 4r_{CH_4}$, and $r_{H_2O} = -r_{CO} - 2r_{CH_4}$.

Based on this, the rates of all species in the system were calculated, as well as the fractional yields (s) for both CH_4 and CO from CO_2 were calculated. Results can be seen in the table below.

Table 1. The rates of all species in CO_2 methanation and the fractional yields for CH_4 and CO from CO_2.

P_{H2}	P_{CO2}	r_{CH4}	r_{CO}	r_{CO2}	r_{H2}	r_{H2O}	$s_{CH4/CO2}$	$s_{CO/CO2}$
0.018	0.250	0.561	0.025	-0.587	-2.271	-1.148	0.957	0.043
0.024	0.250	0.901	0.261	-1.162	-3.865	-2.063	0.775	0.224
0.031	0.250	1.389	0.700	-2.089	-6.254	-3.477	0.665	0.335
0.037	0.250	1.759	0.233	-1.992	-7.268	-3.751	0.883	0.117

As can be seen, $s_{CH4} > s_{CO}$ for the entire range of feed compositions, indicating that the rate of formation of CH_4 production and the selectivity of CH_4 production from CO_2 are higher than that for CO.

In the absence of experimental data to specifically determine each of the rate constants, the relative production or consumption rates of each species can be qualitatively examined to rationally determine the potential rate limiting step of this reaction system.

Stochiometric analysis for the reaction pathway depicted in Fig. 6. produced a formula matrix that was inconsistent and underdetermined and therefore unsolvable. In order to remove these mathematical restrictions, steps (2) and (3) above can be combined to approximate a hybrid step that encompasses both the formation of the surface bound formate, and its subsequent combination with a surface hydride to produce a surface-bound formic acid molecule. The reaction network representing this approximated system then becomes:

(1) $CO_{2(g)} \xrightarrow{k_1} {*CO_2}$

(2) $*CO_2 + *2H \xrightarrow{k_2} *HCOOH$

(3) $*HCOOH \xrightarrow{k_3} CO_{(g)} + H_2O$

(4) $*HCOOH + 6*H \xrightarrow{k_4} CH_{4(g)} + 2H_2O$

Stoichiometric analysis of relationships between species for the reaction system: $r_{*CO2} = r_{CO2} - 3r_{CH4}$, $r_{*H} = -r_{H2O} + r_{CO} - 2_{CH4}$, and $r_{*HCOOH} = r_{H2O} + 2r_{CH4}$.

P_{H2} (atm.)	P_{CO2} (atm.)	r_{CH4}	r_{CO}	r_{CO2}	r_{H2}	r_{H2O}	r_{*CO2}	r_{*H}	r_{*HCOOH}
0.018	0.250	0.562	0.025	-0.587	-2.271	-1.148	-2.271	0.050	-0.025
0.024	0.250	0.901	0.261	-1.162	-3.865	-2.063	-3.865	0.522	-0.261
0.031	0.250	1.389	0.700	-2.089	-6.254	-3.477	-6.254	1.400	-0.700
0.038	0.250	1.759	0.233	-1.992	-7.268	-3.751	-7.268	0.467	-0.233

Looking at these results, we can make the following observations: as P_{H2} in the system increases,

- CH_4 is produced at a faster rate
- CO_2 is consumed faster
- H_2 is consumed faster
- $*CO_2$ is consumed faster
- *H species are produced at the same rate
- CO is produced at the same rate, and
- *HCOOH is consumed at the same rate

In principle, increasing the concentration of H_2 in the feed should, by Le Chatelier's principle, push the equilibrium of reactions (1) to (4) towards their respective product production when the P_{H2} in the feed is increased.

As can be seen from the rate results in the table above, *H is produced at the same rate when P_{H2} in the feed is increased, therefore the generation of *H is not influenced by the increased concentration of H_2 in the system. This could be because the following reaction step (3), the consumption of *HCOOH is also unchanged by the increased concentration of H_2 in the feed, suggesting that the reaction system is "held up" by reaction (4), and the catalyst surface sites are not being vacated by *HCOOH at a rate that would make the surface sites available for the faster generation of *H species.

In contrast, the rate of CH_4 production increases as P_{H2} increases. This is in line with the expected equilibrium shift as a result of Le Chatelier's principle for the overall methanation reaction. We can also eliminate reaction (1) as the potential RDS as $*CO_2$ species are shown to be consumed faster as P_{H2} is increased.

Based on this analysis, we postulate that the RDS for CH_4 production from CO_2 is the final step in which

*HCOOH reacts with a cluster of surface hydrides to desorb as $CH_{4(g)}$.
