# Supplementary material for: Nickel@Siloxene catalytic nanosheets for high-performance CO2 methanation
Source: Nat Commun. 2019 Jun 13;10:2608. doi: 10.1038/s41467-019-10464-x (PMC6565710; doi:10.1038/s41467-019-10464-x)
Supplement: Supplementary file 2 — Description of Additional Supplementary Information [file 41467_2019_10464_MOESM2_ESM.pdf]

Description of Additional Supplementary Files

File Name: Supplementary Movie 1
Description: Volume rendering of 3D reconstruction of a particulate of Ni@SiXNS-EtOH
